# Acute multisystemic thromboembolism manifesting as cerebral infarction, myocardial injury, and quadriparesis: a novel triad of idiopathic hypereosinophilic syndrome

**DOI:** 10.3389/fmed.2025.1685212

**Published:** 2025-11-28

**Authors:** Yong Bai, Ruiling Shang, Minglang Liao, Yanrong Sun

**Affiliations:** Intensive Care Unit, Renmin Hospital, Hubei University of Medicine, Shiyan, Hubei, China

**Keywords:** hypereosinophilic syndrome, eosinophil-mediated microvascular thrombosis, acute myocardial infarction, cerebral infarction, quadriparesis, corticosteroid therapy, idiopathic hypereosinophilic syndrome, multisystemic thromboembolism

## Abstract

**Background:**

Hypersegmented Eosinophil Syndrome (HES) is a rare myeloproliferative condition with several clinical symptoms. Idiopathic HES presenting with acute, contemporaneous multisystem thrombotic episodes is extremely unusual, making clinical detection difficult and increasing the risk of misdiagnosis.

**Case presentation:**

This paper describes the case of a 69-years-old male patient with a history of coronary heart disease and hypertension, who was admitted for “dizziness accompanied by discomfort in the left shoulder and precordial region.” The patient was initially diagnosed with posterior circulation ischemia, but quickly developed a clinical trial of acute cerebral infarction (confirmed by cranial MRI), non-ST-segment elevation myocardial infarction (confirmed by progressively rising cardiac markers and ECG), and acute quadriplegia (muscle strength 0–3). Laboratory tests revealed significant peripheral blood eosinophilia (43.4%). Coronary CT scans ruled out acute in-stent restenosis. Bone marrow aspiration and genetic testing ruled out other clonal anomalies, resulting in a diagnosis of “idiopathic hypereosinophilic syndrome.”

**Discussion:**

This case report describes a novel clinical situation in which an acute thromboembolic triad (cerebral infarction, cardiac damage, and quadriplegia) is the first presentation of idiopathic HES. It demonstrates that eosinophil-mediated hypercoagulability is the primary pathogenic mechanism behind multisystem embolism.

**Conclusion:**

Prompt blood tests to assess eosinophil counts remain critical for early identification of HES, especially in the absence of usual clinical history. Early and strong glucocorticoid treatment efficiently reduces eosinophil levels and improves prognosis.

## 1 Introduction

A diverse collection of illnesses known as hypereosinophilic syndrome (HES) is typified by end-organ damage and persistent eosinophilia (≥1.5 × 10^9^/L) (1). The co-occurrence of acute cerebral infarction, non-ST-elevation myocardial infarction (NSTEMI), and rapidly progressive quadriparesis due to eosinophil-mediated microvascular thrombosis is extremely uncommon and difficult to diagnose, even though cardiac and neurological involvement are acknowledged complications (2).

Endothelial damage, hypercoagulability, and microthrombosis are encouraged by the cytotoxic granules (such as major basic protein and eosinophil cationic protein) and procoagulant substances released by eosinophils (3–5). Myocarditis and intracavitary thrombi are examples of cardiac symptoms, whereas encephalopathy or embolic stroke are examples of neurological aftereffects. But there is little information on the acute triad of stroke, NSTEMI, and flaccid paralysis with increasing eosinophilia, especially in myeloproliferative-variant or idiopathic HES lacking common driver mutations (e.g., *FIP1L1-PDGFRA*, PDGFRB) (6).

Here, we describe a 69-years-old man who had a history of hypertension and coronary artery disease (CAD) and who came in with acute limb weakness, vertigo, and chest pain. Progressive eosinophilia (43.4%) with increasing troponin, CK-MB, and neuromuscular degeneration led to a unifying diagnosis of HES, despite the initial examination suggesting posterior circulation ischemia and NSTEMI. Targeted corticosteroid treatment quickly restored eosinophil numbers and cured paralysis in spite of negative genetic investigations for classic HES mutations, underscoring the crucial role that early immunosuppression plays in eosinophil-mediated multiorgan failure. The need of eosinophil-directed therapy when thrombotic microangiopathy is suspected is highlighted by this instance, which also highlights HES as a masquerader of acute cardiocerebrovascular disorders.

## 2 Case presentation

On March 27, 2025, a 69-years-old male patient was admitted due to “dizziness accompanied by discomfort in the left shoulder and precordial region for 1 day.” The patient had a rapid start of dizziness and a spinning sensation 1 day before admission (March 26, 2025), which got worse with movement. This was accompanied by self-reported “numbness in the limbs” and pain in the precordial area and left shoulder. There were no other clinical signs or symptoms. The discomfort in the left shoulder and left precordial area did not much reduce after rest, however the dizziness did slightly. The patient came to our hospital in search of additional assessment and care. The patient was initially admitted to the outpatient department with a diagnosis of “posterior circulation ischemia.” The patient’s mental state, eating, sleep patterns, and bowel and urine functions have all been normal from the beginning of the illness. Body weight has not changed significantly, but physical strength has diminished.

Anterior descending coronary artery stenting was performed during PCI due to a 5-years history of coronary artery disease. Aspirin 100 mg once day and Rosuvastatin Calcium 10 mg once daily are the medications that he currently use on a regular basis. Over 10 years of history of hypertension; currently using amlodipine benzenesulfonate tablets 5 mg once day and metoprolol tartrate tablets 25 mg once daily orally as part of a routine treatment regimen. Has a lengthy history of drinking and smoking; denies any additional medical history.

Physical examination: blood pressure of 134/82 mmHg, temperature of 36.3 °C, pulse rate of 75 beats per minute, and respiration rate of 19 breaths per minute. During the examination, the patient is cooperative, focused, and has clear communication. All limbs have reduced muscle strength, normal muscle tone, and symmetrical bilateral tendon reflexes. A physical check revealed no other noteworthy anomalies. Initial Admission Diagnosis: (1) Posterior circulation ischemia. (2) After PCI for coronary atherosclerotic heart disease. (3) Hypertension, Grade 2 (extremely high risk).

**FIGURE 1:**
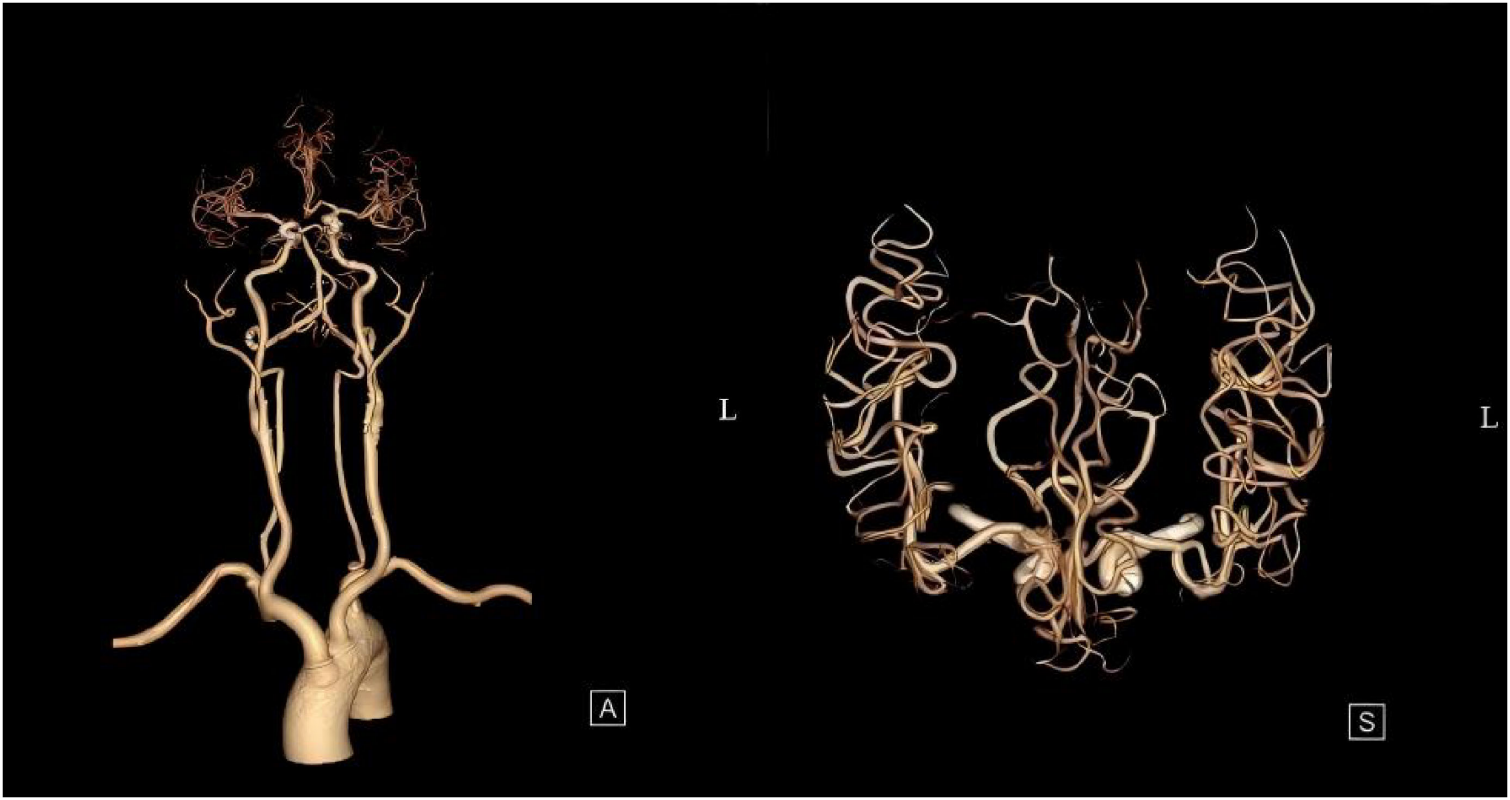
Cranial vascular CTA suggestive of cerebral atherosclerosis.

**FIGURE 2:**
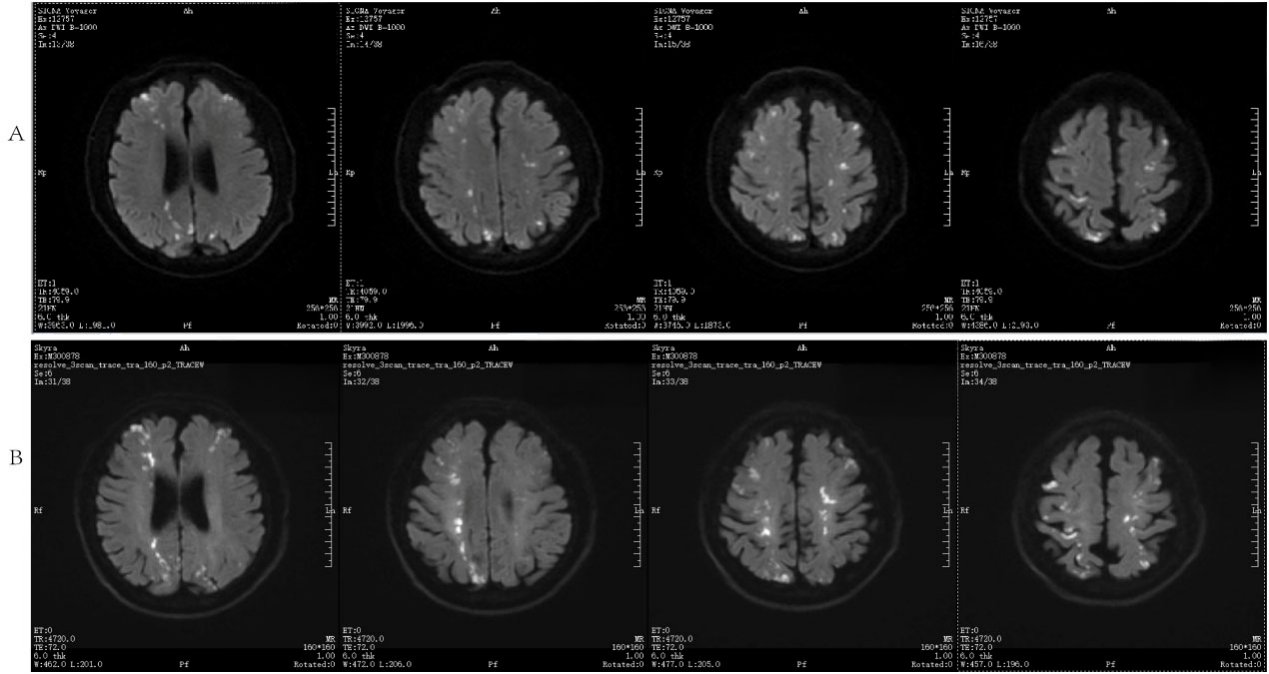
DWI and cranial MRI **(A)** inspected on March 27, 2025, **(B)** analyzed on April 1, 2025.

**FIGURE 3:**
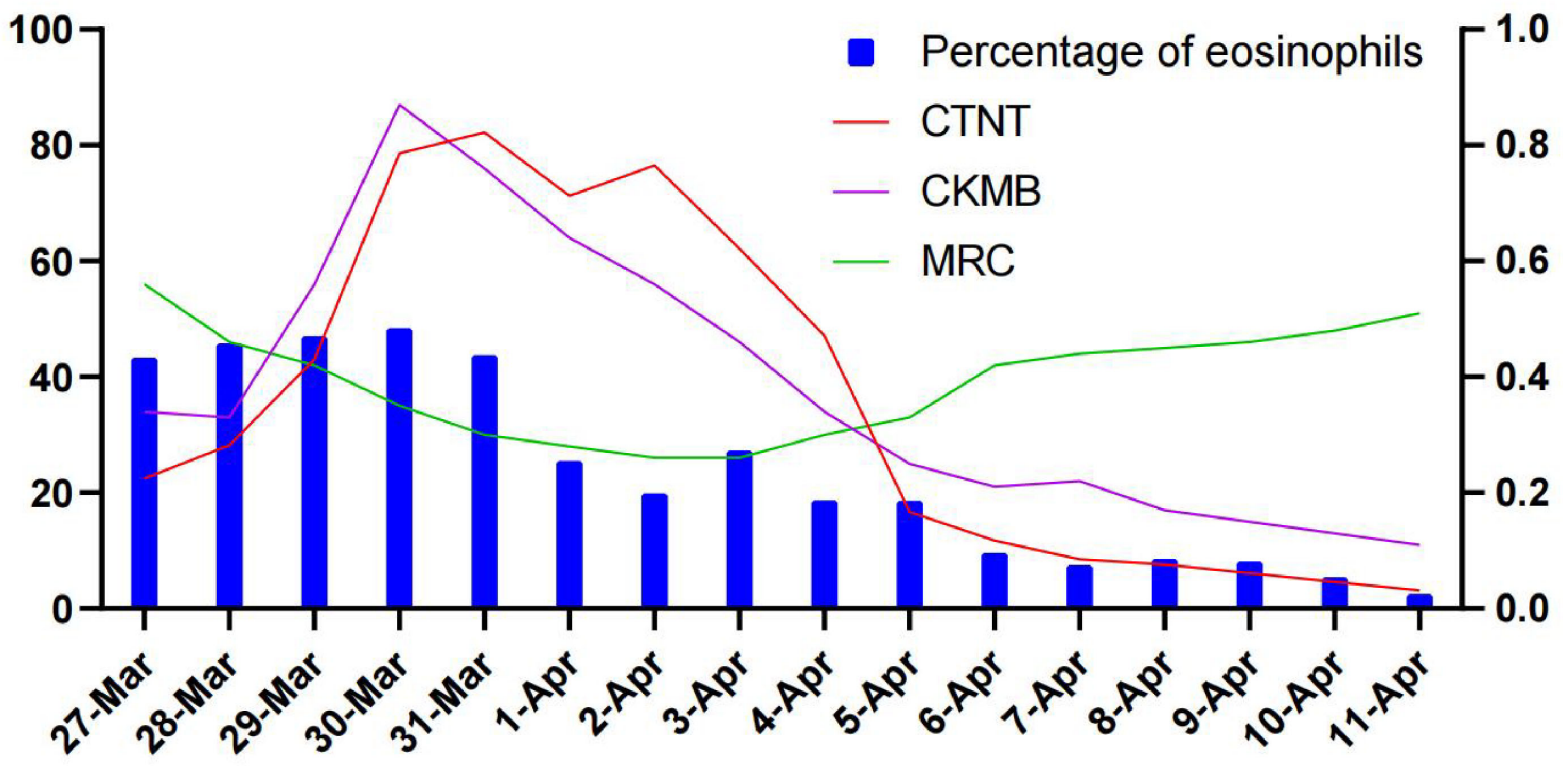
Percentage of eosinophils (%), Ardiac troponin T (CTNT) (ng/ml), Creatine Kinase-MB (CKMB) (U/L), Medical Research Council (MRC) muscle strength score.

**FIGURE 4:**
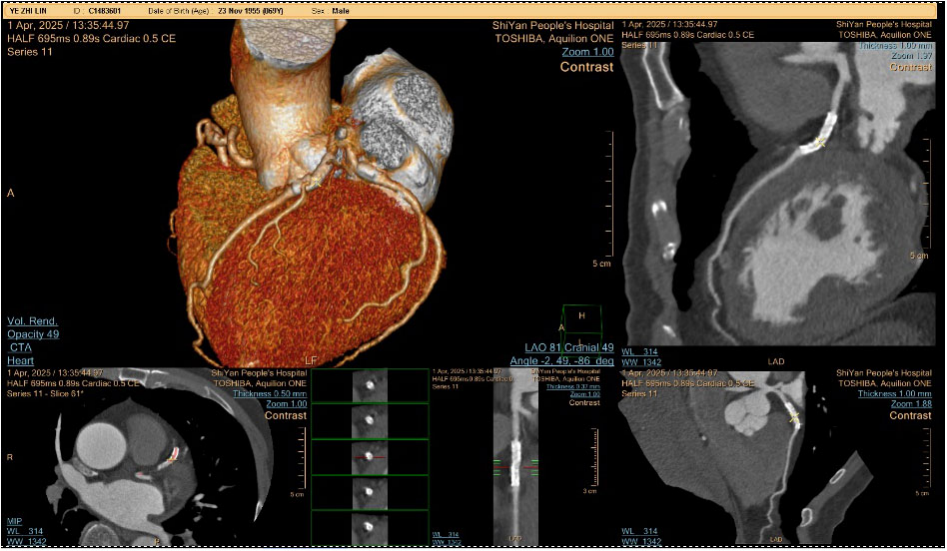
Coronary CTA examination.

**FIGURE 5:**
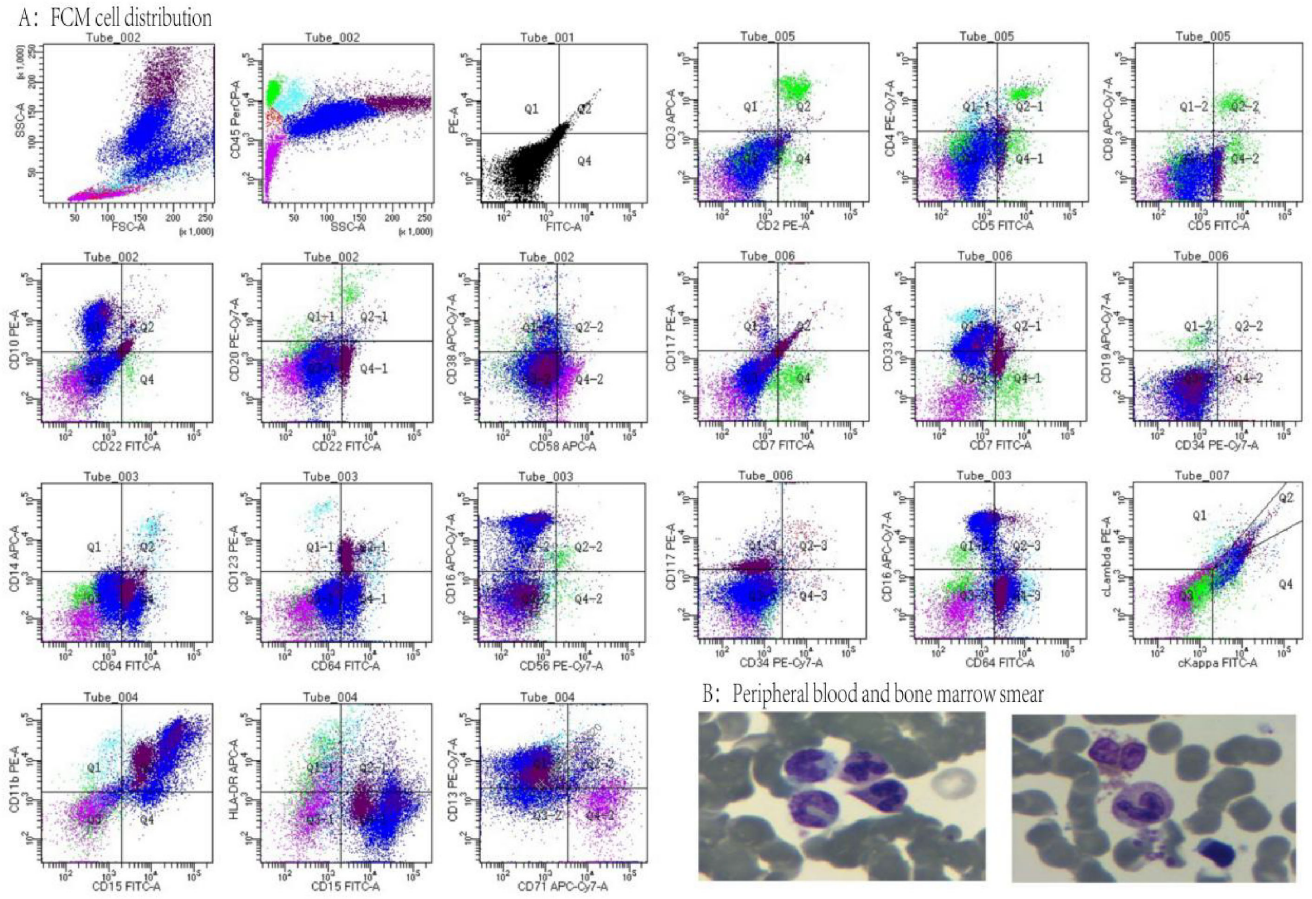
**(A)** Flow cytometry cell distribution, lymphocytes (green) 7%, primitive zone cells (red) 1%, monocytes (sky blue) 2%, eosinophils (dark red) 17.5%, neutrophils (blue) 59%, nucleated red zone cells (purple) 13.5%. **(B)** Peripheral blood and bone marrow smears.

The patient was admitted to the hospital, and a cranial vascular CTA examination revealed cerebral atherosclerosis without obvious vessel stenosis (see Figure 1); a cranial MRI and DWI examination revealed bilateral cerebellar, bilateral frontal-parietal-temporo-occipital, and semiovoid central region infarcts (see Figure 2A); troponin levels were 0.224 ng/ml [ref (ref), 0.0–0.08 ng/ml], Creatine kinase isoenzyme 34 U/L [ref, 0.0–24 U/L], N-terminal brain natriuretic peptide precursor (BNP) 1324 pg/ml [ref, 0.0–300 pg/ml], and an electrocardiogram that suggested an ST-segment abnormality. Cardiovascular consultation was sought to rule out a non-ST-segment elevation myocardial infarction, and antiplatelet aggregation and lipid-modulating therapy were administered. The percentage of eosinophils was 43.4% [ref, 0.4%–8%], and tests for coagulation function, lipids, and liver and renal function revealed no appreciable abnormalities. Two days later, the patient developed severe precordial discomfort and dyspnea. Concurrently, limb muscle strength decreased significantly. On March 31, a physical examination revealed grade 3 muscle strength in the left upper limb, 0 in the right upper limb, and grade 2 muscle strength in both bottom limbs. Figure 3 depicts muscle strength scores provided by the Medical Research Council (MRC). Troponin, creatine kinase isoenzyme, and eosinophil percentage were all steadily declining compared to the prior time frame. As shown in Figure 3, the acidophilic fraction increased steadily. He was moved to the critical care unit (ICU) on March 31 in order to receive correctional treatment. According to a perfect coronary CTA examination, the coronary artery wall had many segmental mixed plaque formation, mild stenosis in the lumen, and an anterior descending branch of the middle section of the stent, which was about 2.3 cm long. The stent did not exhibit any visible fractures or restenosis symptoms, as shown in Figure 4. Bilateral cerebellum, bilateral frontal-parietal-temporo-occipital and semiovarian central area of several spots, and a tiny patchy DWI high signal, measuring between 2 and 18 mm (slightly larger in extent than the previous MR 2025.03.27), were all suggested by the review of the cranial MRI test (see Figure 2B). On April 1, tests revealed a considerably higher eosinophil level. Eosinophilia was discovered following a consultation with the hematology department. To decrease eosinophils, methylprednisolone sodium succinate was administered first, followed by clopidogrel to limit platelet aggregation and low molecular weight heparin for anticoagulation. A bone marrow aspiration was performed to assess flow cytometry, FISH, and fusion genes. Figure 5A shows the flow cytometry results for April 5: an elevated eosinophil percentage. According to the bone marrow aspiration report, there were more eosinophils in the bone marrow and peripheral blood images. Figure 5B shows an increase in eosinophils. Fusion gene screening revealed no results for PCM1:JAK2, ETV6:JAK2, FLT3, ETV6:ABL1, and BCR:JAK2. FISH probe analysis: FGFR1, PDGFRA, and PDGFRB: negative. Low molecular heparin anticoagulation, clopidogrel antiplatelet aggregation, and eosinophils were all reduced by methylprednisolone sodium succinate. Eosinophil counts in the patient progressively returned to normal. The patient’s limb muscle strength improved between 4 and 11; both upper and lower limb muscular strength were grade 3 and grade 4, respectively. He was sent to the rehabilitation department for therapy, and on April 28, he was released from the hospital on June 1. The outpatient follow-up assessment showed that the patient’s limb muscle strength had restored to normal.

## 3 Discussion

Although hypereosinophilic syndrome (HES) can appear as acute cardiocerebrovascular syndromes (7, 8), it is not commonly known to manifest with flaccid quadriparesis, non-ST-elevation myocardial infarction (NSTEMI), and cerebral infarction. Three important clinical lessons can be learnt from our case: (1) eosinophil-mediated microvascular thrombosis as a common mechanism for multiorgan failure; (2) strong “diagnostic inertia” may result from pre-existing atherosclerotic disease, delaying the identification of HES; and (3) the necessity of immediate eosinophil-directed treatment even in the face of negative molecular stratification.

### 3.1 Pathophysiological mechanisms of the “thrombotic storm”

The abrupt neurological, cardiac, and neuromuscular deterioration that our patient is experiencing is consistent with the hypereosinophilia-induced prothrombotic condition. Release of eosinophils: procoagulant factors that encourage fibrin deposition (9), such as von Willebrand factor and tissue factor (10, 11); cytotoxic granules that cause endothelium apoptosis, such as eosinophil peroxidase, a key basic protein; Vasoactive mediators that cause vasospasm include leukotrienes and platelet-activating factor (12).

This “thrombotic storm” caused microinfarcts in the myocardial (troponin increase without severe coronary stenosis), skeletal muscle microvasculature (acute quadriparesis with MRC 0–2), and brain (MRI-confirmed widespread DWI lesions). Despite patent coronary stents, increasing troponin/BNP indicated that eosinophilic myocarditis and microthrombi were most likely the cause of the cardiac involvement. Different from large-vessel stroke, neuromuscular paralysis was a reflection of peripheral nerve/muscle microvascular ischemia. The effectiveness of glucocorticoids in lowering eosinophil levels has been demonstrated by studies (13–15). This mechanism explains the rapid resolution of tetraplegia caused by eosinophil depletion brought on by glucocorticoid usage.

Clarifying the pathological core of this condition also requires differentiating eosinophilic vasculitis (such as eosinophilic granulomatous polyangiitis, EGPA) from eosinophilia syndromes related to other diseases. EGPA symptoms include asthma, allergic rhinitis, eosinophilia, and neuropathy. Its vasculitic character results in direct eosinophil infiltration of the vascular wall, which causes necrotizing inflammation (16). However, this case lacks a history of asthma, typical systemic vasculitis symptoms (such as renal involvement or purpura), and supporting serological markers (such as ANCA). More crucially, the extensive microinfarctions seen in our patient are more consistent with a “embolic storm” caused by eosinophil-mediated systemic hypercoagulability than with focal inflammatory damage of the arterial wall. This is also corroborated by the lack of typical vasculitic characteristics on both coronary and cerebral angiography. As a result, we attribute the pathogenesis of this case predominantly to idiopathic HES-related eosinophil toxicity and a prothrombotic condition, rather than a conventional vasculitic process. This divergence in mechanism is critical since it emphasizes the importance of fast eosinophil suppression (immunosuppression) in management alongside anticoagulant medication.

### 3.2 Diagnostic challenges and pitfalls

Evaluation was first directed toward atherosclerotic complications (“posterior circulation ischemia” and “NSTEMI”) due to our patient’s history of CAD and hypertension. Three results, however, contradicted this: Coronary CTA: Myocardial damage is not caused by plaque rupture, rather mild stenosis; Temporal correlation: eosinophil increase (peak 43.4%) was accompanied by rising troponin/CK-MB, not ECG/angiographic abnormalities; Neurological paradox: The stable MRI infarct burden was not matched by progressive quadriparesis. This emphasizes that even in cases of established CAD, HES can mimic acute coronary syndromes, making the eosinophil count a “vital sign” in thrombotic presentations. Despite thorough FISH/RT-PCR screening, the lack of *FIP1L1-PDGFRA*, PDGFRB, FGFR1, and JAK2 fusions indicates that this is either idiopathic or myeloproliferative HES. This has two meanings: Diagnostic difficulty: In molecular-negative HES, such severe presentations are uncommon.

In this example, extensive FISH and RT-PCR screening failed to identify known fusion genes such as *FIP1L1-PDGFRA*, PDGFRB, FGFR1, and JAK2. This negative finding has important consequences for both diagnosis and treatment. Diagnostically, it efficiently excludes myeloid tumors caused by the aforementioned unique gene rearrangements. These disorders are well identified primary hematologic malignancies that respond effectively to imatinib-targeted therapy. However, a negative result does not rule out all possible clonal origins. For example, it is unable to detect lymphocytic HES variations caused by aberrant T-cell clones, as well as other unusual genetic changes that are currently unknown to current technology. Therefore, given the existing diagnostic framework, this patient is classed as idiopathic HES. This classification means that glucocorticoids are the indisputable first-line treatment option.

### 3.3 A novel perspective on HES-associated paralysis

Therapeutic opportunity: Corticosteroids continue to be the first-line treatment, resulting in quick organ recovery and eosinophil depletion (eosinophils returned to normal within 10 days, and paralysis was gone by Day 40). Idiopathic HES may need long-term IL-5 inhibition (e.g., mepolizumab) to maintain remission, in contrast to imatinib-sensitive HES, where targeted therapy prevents relapse (17). This approach is necessary in the event that steroids are ineffective or relapse occurs. This patient’s symptoms improved quickly after glucocorticoid medication, with no steroid dependence or recurrence. Otherwise, adding moprolizumab is the chosen escalation treatment strategy.

In HES, flaccid paralysis is usually caused by eosinophilic neuropathy or embolic stroke (18). Our case offers fresh perspectives: The microvascular cause: Despite stable brain infarcts, paralysis worsened; electromyography, which was regrettably not done, might have revealed axonal degeneration from microthrombotic ischemia; Response to corticosteroids: Motor recovery took 2 weeks longer than cardiac recovery, indicating that neuromuscular microvasculature repair takes time. In contrast to cerebral embolism or Churg-Strauss vasculitis, We believe that eosinophilic microvascular ischemic neuropathy is a significant and treatable cause of acute critical paralysis in HES, which doctors should distinguish from cerebral embolism or vasculitic neuropathy.

### 3.4 Clinical recommendations and future directions

Future directions and some clinical advice can be found in this case report. An algorithm for diagnosing thrombosis linked to HES: Every patient with “cryptogenic” multiorgan thrombosis should have their eosinophil count measured; When HES is suspected, do nerve conduction testing (if paralyzed) and cardiac MRI (for myocardial inflammation). Urgency for treatment: If HES is likely, start corticosteroids very once because delaying treatment could cause irreversible organ damage. Anticoagulation, such as heparin, is an adjuvant but insufficient treatment without immunosuppression. Frontiers of research: Check molecular-negative HES for new mutations (e.g., STAT5B, JAK1); investigate IL-5/IL-3/IL-13 inhibition for steroid-refractory microvascular damage.

## 4 Conclusion

By showing that eosinophil cytotoxicity can concurrently target the brain, heart, and peripheral neuromuscular system–even disguising itself as atherosclerotic emergencies–this example reinterprets the parameters of HES-induced thrombotic microangiopathy. Eosinophil depletion is the cornerstone of treatment, as evidenced by the quick recovery of cardiac damage and quadriparesis with corticosteroids. Our patient’s negative genetics serve as a reminder to clinicians in the age of molecular diagnostics that when eosinophilia orchestrates a thrombotic crisis, prompt empirical immunosuppression can save lives.

## Data Availability

The raw data supporting the conclusions of this article will be made available by the authors, without undue reservation.
